# Primary Diffuse Large B-cell Lymphoma of the Urinary Bladder: A Case Report and Narrative Review of Treatment Modalities and Clinical Outcomes

**DOI:** 10.7759/cureus.115437

**Published:** 2026-08-30

**Authors:** Ramyar M Abdullah, Kelvin B Raybon, Brian Stisser, Christopher J Wenzinger

**Affiliations:** 1 Internal Medicine, Augusta Health, Fishersville, USA; 2 Cancer Center, Augusta Health, Fishersville, USA; 3 Urology, Augusta Health, Fishersville, USA; 4 Pathology, Augusta Health, Fishersville, USA

**Keywords:** de-escalated therapy, diffuse large b-cell lymphoma, extranodal lymphoma, frailty, primary bladder lymphoma, review, rituximab therapy

## Abstract

Primary diffuse large B-cell lymphoma (DLBCL) of the urinary bladder is a rare extranodal lymphoma, representing a very small proportion of both bladder malignancies and extranodal lymphomas. We report a 73-year-old, chronically institutionalized woman with Eastern Cooperative Oncology Group (ECOG) 3-4 who presented with abdominal/flank pain, fever, hydronephrosis, and polymicrobial urosepsis. A bladder mass was detected incidentally on CT and subsequently confirmed by cystoscopy and transurethral resection of bladder tumor (TURBT). Histopathology showed a CD20-positive, CD5/CD10-negative large B-cell lymphoma (BCL) with Ki-67 of 50% (focally up to 80%), multiple myeloma oncogene (MUM)-1 positivity, and an isolated BCL6 rearrangement without concurrent myelocytomatosis oncogene (MYC) or BCL2 abnormalities, consistent with a high-grade, non-GCB DLBCL. Given her frailty and comorbidities, she was treated with rituximab monotherapy followed by involved-site radiotherapy instead of anthracycline-based chemoimmunotherapy, achieving marked metabolic and anatomic regression of the bladder lesion on positron emission tomography (PET)/CT. We present a narrative review of the literature on primary bladder DLBCL, highlight diagnostic and therapeutic considerations in the context of severe frailty, and discuss how de-escalated, organ-preserving strategies may be appropriate for selected patients.

## Introduction

Diffuse large B-cell lymphoma (DLBCL) is the most common aggressive subtype of non-Hodgkin lymphoma (NHL), representing approximately 30% of NHL cases worldwide [1]. Gene-expression profiling separates DLBCL into germinal center B-cell (GCB)-like and activated B-cell (ABC)-like/non-GCB molecular groups, each characterized by distinct oncogenic pathways and prognostic behavior [2,3]. Although extranodal presentation is common, occurring in roughly 25-40% of lymphomas, primary involvement of the urinary bladder is exceptionally uncommon and accounts for fewer than 1% of bladder malignancies and approximately 0.2% of extranodal lymphomas [4,5].

Most primary bladder lymphomas are mucosa-associated lymphoid tissue (MALT) lymphomas, while DLBCL accounts for only about 20-30% of reported cases and represents the more aggressive histologic subtype [5,6]. Since the first description of bladder lymphoma in the late nineteenth century, fewer than 150 primary bladder lymphoma cases have been reported, and primary bladder DLBCL remains particularly rare [7,8]. Patients most often present with painless gross hematuria or irritative lower urinary tract symptoms (LUTS), but the diagnosis can be obscured by concurrent infection, obstruction, or inflammatory bladder disease [5-9].

Because prospective trials and disease-specific guidelines are lacking, treatment is usually extrapolated from systemic DLBCL management. For medically fit patients, rituximab + cyclophosphamide + doxorubicin + vincristine + prednisolone (R-CHOP)-based chemoimmunotherapy, with or without consolidative radiotherapy, remains the most commonly reported approach [6,9,10].

We report a case of localized primary bladder DLBCL in a profoundly frail, chronically institutionalized older woman who was not a candidate for anthracycline-based treatment and who achieved meaningful disease control with rituximab monotherapy followed by involved-site radiotherapy. We also present a narrative review summarizing the published experience with treatment modalities and outcomes in primary bladder DLBCL, drawing on cases identified through a targeted literature search (search strategy and eligibility criteria described in the Literature Review section).

## Case presentation

The patient was a 73-year-old woman with an Eastern Cooperative Oncology Group (ECOG) performance status of 3-4, residing in a chronic care facility for 12 years prior to presentation. Her comorbidities included traumatic brain injury with organic brain syndrome, chronic seizure disorder, non-ambulatory status since age 61, ischemic stroke with left-sided hemiparesis, and chronic urinary incontinence. She presented with polymicrobial urosepsis due to *Escherichia coli* and *Proteus mirabilis*. Contrast-enhanced CT of the abdomen and pelvis revealed a focal left bladder wall mass with paravesical soft-tissue inflammatory changes and left-sided hydronephrosis and hydroureter, without lymphadenopathy (Figure 1). She was admitted, treated with antibiotics, and discharged after two days with outpatient urology follow-up.

**Figure 1:**
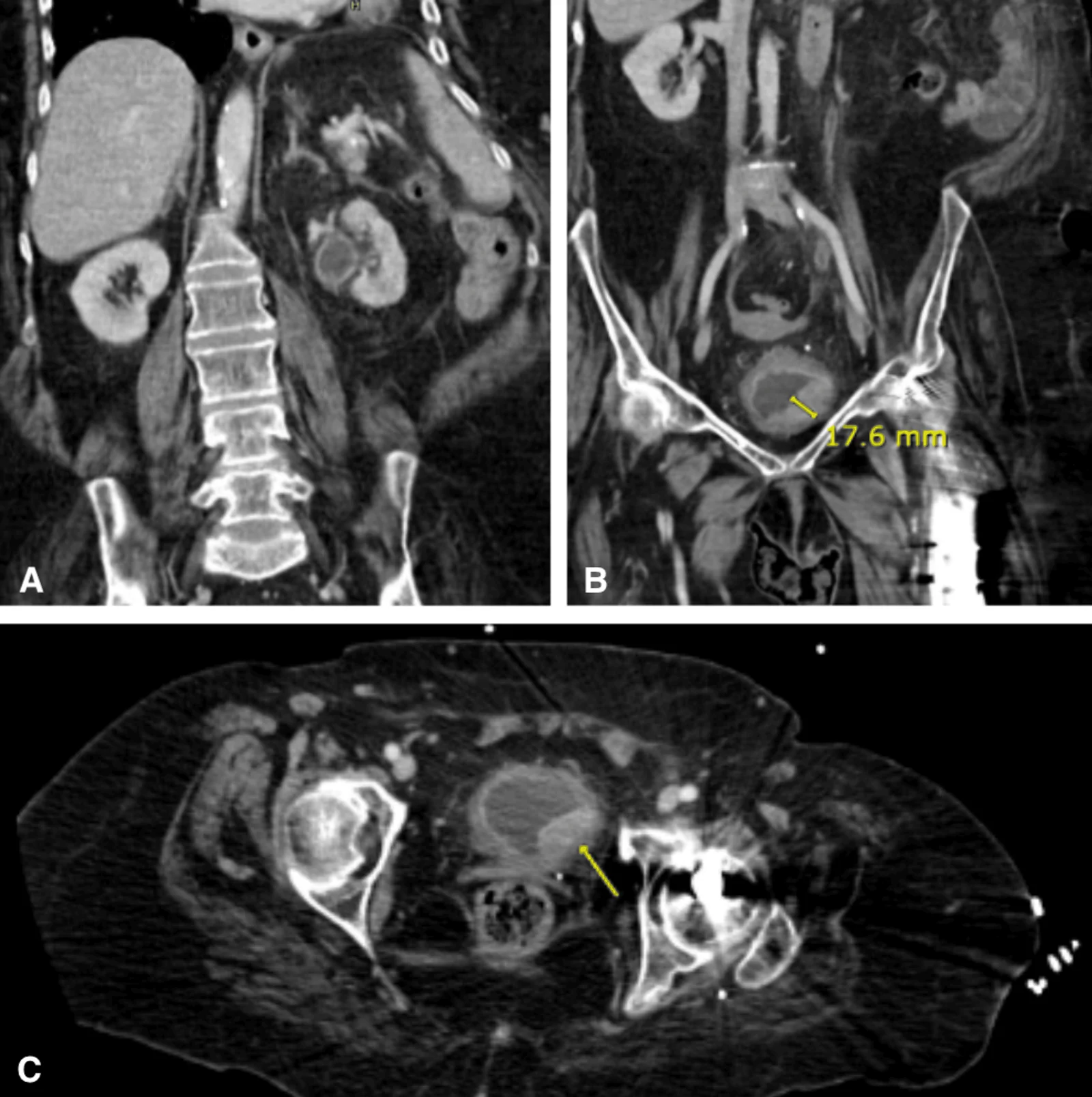
Contrast-enhanced CT of the abdomen and pelvis obtained prior to TURBT (A) Coronal CT image demonstrating left-sided hydronephrosis; (B, C) Coronal and axial images, respectively, revealing a focal mural mass involving the left urinary bladder wall (17.6 mm in greatest transverse dimension; yellow line), with associated paravesical soft-tissue inflammatory changes (yellow arrow). Metallic streak artifacts are attributable to an in situ compression screw at the left femoral head and neck. TURBT: Transurethral resection of bladder tumor

The patient underwent cystoscopy with bilateral retrograde pyelograms, which revealed the right side to be essentially normal; the left side showed irregularity of the renal pelvis and calyces with no obvious filling defect. There was a mass-like effect along the left bladder wall just proximal to the bladder neck with an edematous appearance; it did not appear typical for transitional cell carcinoma. The lesion was resected down into what appeared to be atypical muscle. There appeared to be tumor nests within the tissue. This was a thorough but likely incomplete resection. A postprocedural retrograde pyelogram showed no extravasation. Positron emission tomography (PET)/CT performed after transurethral resection of bladder tumor (TURBT) and prior to initiation of systemic therapy and radiotherapy demonstrated decreased conspicuity of asymmetric left posterior bladder wall thickening and an asymmetric linear uptake along the right posterior bladder wall (Figure 2). This finding most likely reflects residual disease extending toward the midline and right side of the bladder that was not fully appreciated on the pre-procedural CT.

**Figure 2:**
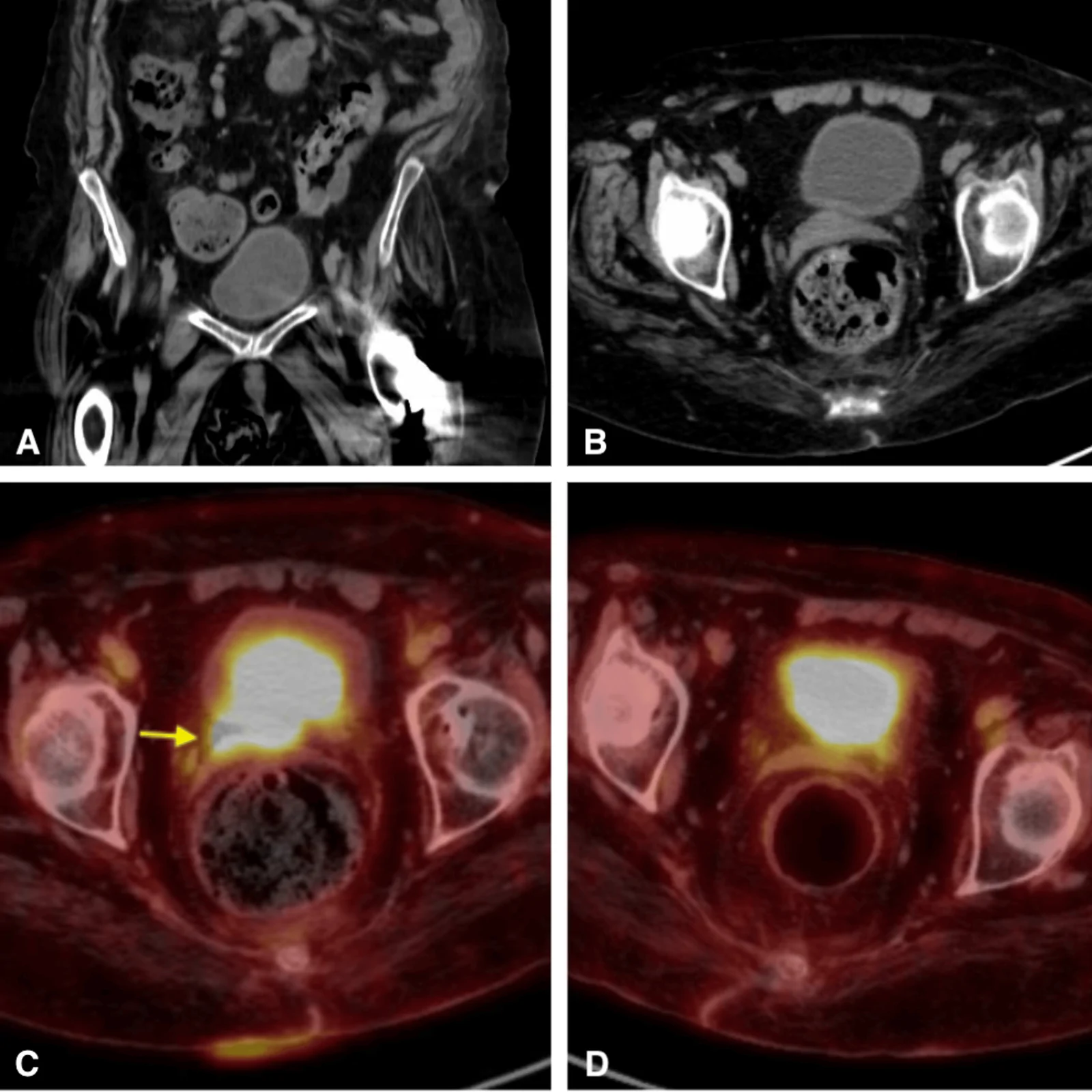
Pre- and post-treatment FDG-PET/CT of primary bladder DLBCL (A, B) Axial CT images demonstrating decreased conspicuity of asymmetric left posterior bladder wall thickening with subtle urothelial irregularity; (C) Axial fused FDG-PET/CT image demonstrating subtle linear asymmetric FDG uptake at the midline and right of midline along the posterior bladder wall (maximum SUV 11; yellow arrow); (D) Axial fused FDG-PET/CT image obtained following completion of systemic therapy and radiotherapy, demonstrating further interval reduction in lesion size and FDG avidity at the posterior bladder. Assessment of residual metabolic activity is limited by physiologic urinary tracer excretion; however, overall imaging appearances are improved, with no evidence of hypermetabolic disease at additional sites. FDG: Fluorodeoxyglucose; PET: Positron emission tomography; DLBCL: Diffuse large B-cell lymphoma; SUV: Standardized uptake value

Pathology revealed a B-cell lymphoma (BCL), CD5-negative and CD10-negative, with large-cell zones suggesting a high-grade lymphoma (Figure 3). The histopathology is summarized in Table 1, and baseline laboratory findings are summarized in Table 2. Due to comorbidities and functional status, the patient was not a candidate for anthracycline-based chemotherapy, so she was started on rituximab 375 mg/m2 IV for four-weekly doses and involved-site radiation therapy. Treatment was delivered with a volumetric modulated arc therapy (VMAT)-based intensity-modulated radiation therapy (IMRT) plan to a total dose of 4,000 cGy in 20 fractions over 27 days, with daily cone-beam CT image guidance. Follow-up PET/CT about three months after finishing treatment showed improved bladder thickening and left hydronephrosis, with interval decrease in mass and fluorodeoxyglucose (FDG) uptake posterior to the bladder, now with very little activity present. Standardized uptake value (SUV) calculation was difficult due to physiologic uptake of the nearby GU tract. She had no clinical evidence of progressive disease, and PET/CT appeared reassuring for no rapidly progressive disease (Figure 2). At follow-up with urology, cystoscopy was deemed unnecessary based on the imaging findings. She remains alive and is residing in a chronic care facility eight months after diagnosis, with ongoing follow-up.

**Figure 3:**
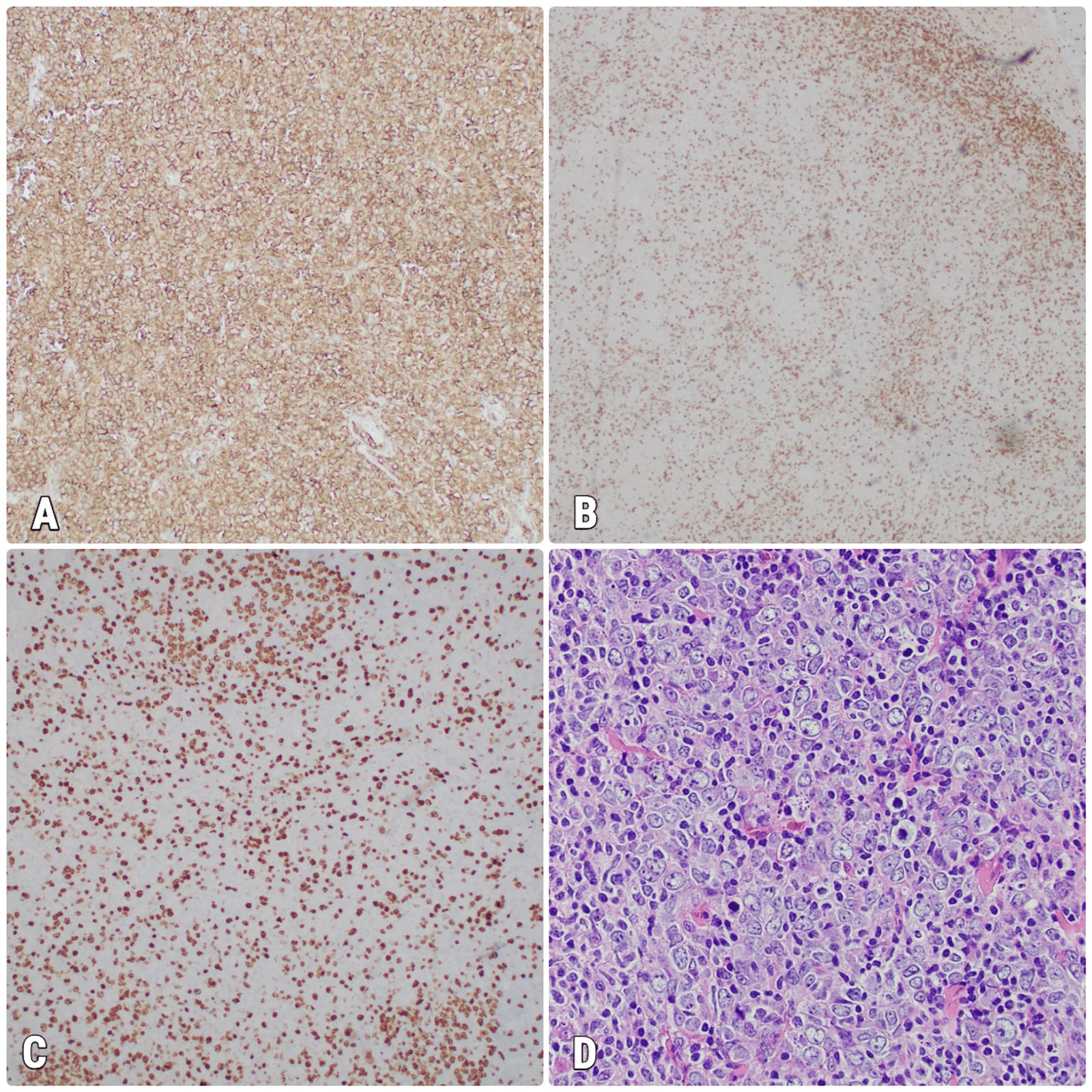
Histopathological and immunohistochemical features of the primary bladder mass (A) Immunohistochemical staining for CD20 demonstrating diffuse, strong membranous positivity throughout the neoplastic lymphoid population, confirming B-cell lineage; (B) CD3 immunostaining is negative in the neoplastic large B-cell population, with staining limited to scattered background/reactive T lymphocytes; (C) Ki-67 immunostaining demonstrating a markedly elevated proliferative index among the neoplastic cells, consistent with the aggressive clinical behavior of DLBCL; (D) H&E-stained section demonstrates a diffuse, expansile lymphoid infiltrate with a morphologic spectrum ranging from small to intermediate forms to overtly large cells with vesicular nuclei and prominent nucleoli, including discrete zones of diffuse large-cell proliferation. DLBCL: Diffuse large B-cell lymphoma; H&E: Hematoxylin and eosin

**Table 1:** Summary of diagnostic findings BCL: B-cell lymphoma; DLBCL: Diffuse large B-cell lymphoma; MYC: Myelocytomatosis oncogene; MUM: Multiple myeloma oncogene; PAX5: Paired box gene; EBER: Epstein-Barr virus-encoded small RNA; ISH: In situ hybridization; FISH: Fluorescence in situ hybridization

Category	Finding
Disease stage	Stage IE (left urinary bladder, BCL)
Histology	DLBCL
Immunohistochemical stains	CD20+, CD45+, CD3−, CD5−, CD10−, BCL6−, PAX5+ (strong, large cell component); Ki-67: 50% (focal areas up to 80%); MYC: Negative; MUM-1: Partially positive (>30% large cell component); EBER (ISH): Negative
FISH	BCL-6 rearrangement: Positive. MYC (8;14) and BCL-2 (14;18) fusions: Negative. MYC and BCL-2 rearrangements: Negative
Flow cytometry and peripheral blood	Monoclonal B cells: Not detected

**Table 2:** Baseline laboratory findings Laboratory studies were obtained during diagnostic workup, prior to initiation of systemic therapy. H denotes a result above the reference range. WBC: White blood cell count; LDH: Lactate dehydrogenase; HBV: Hepatitis B virus; HCV: Hepatitis C virus; HIV: Human immunodeficiency virus

Parameter	Result	Reference Range
WBC	4.48×10³/mm³	3.5-11.0×10³/mm³
Hemoglobin	11.5 g/dL	11.0-16.0 g/dL
Platelet count	317.7×10³/mm³	140-400×10³/mm³
Total protein	7.5 g/dL	6.0-8.3 g/dL
Creatinine	0.88 mg/dL	0.60-1.20 mg/dL
LDH	124 IU/L	<247 IU/L
β2-microglobulin	4.5 mg/L (H)	0.6-2.4 mg/L (H)
Viral serologies (HBV, HCV, HIV)	All negative/non-reactive	-

## Discussion

The present case illustrates primary stage IE DLBCL of the urinary bladder in a profoundly frail, chronically institutionalized 73-year-old woman whose initial presentation closely mimicked complicated urinary tract infection rather than a bladder malignancy. Her symptoms (flank/abdominal pain, fever, hydronephrosis, and polymicrobial urosepsis) and contaminated urinalysis led first to an infectious diagnosis, with the bladder mass identified incidentally on CT and later confirmed by cystoscopy and TURBT. The tumor showed a non-GCB immunophenotype with CD20 positivity, CD5 and CD10 negativity, and a Ki-67 proliferation index of 50% with focal areas up to 80%, consistent with high-grade aggressive biology.

Most notably, fluorescence in situ hybridization (FISH) demonstrated an isolated BCL6 rearrangement without concurrent myelocytomatosis oncogene (MYC) or BCL2 rearrangement.Although this profile is sometimes informally termed “single-hit,” it is distinct from the WHO-defined category of high-grade BCL with MYC and BCL2 and/or BCL6 rearrangements (double-/triple-hit lymphoma), which specifically requires a concurrent MYC rearrangement; the independent prognostic significance of an isolated BCL6 rearrangement is less well established and should be interpreted with caution [3,11]. The multiple myeloma oncogene (MUM)-1 partial positivity in the large-cell component further supports a non-GCB phenotype, which is generally linked to nuclear factor kappa-light-chain-enhancer of activated B cells (NF-κB) pathway activation and worse outcomes than GCB disease [2,3].

Biochemically, normal lactate dehydrogenase (LDH) and negative peripheral blood flow cytometry favored localized disease, in keeping with Ann Arbor Stage IE. The mildly elevated β2-microglobulin may reflect renal impairment related to long-standing hydronephrosis and recurrent urinary infections rather than high lymphoma burden; baseline glomerular filtration rate and longitudinal renal function data were not available to confirm this interpretation, and a contribution from tumor burden cannot be entirely excluded. Negative viral serologies argue against an immunodeficiency-associated process. The TURBT was necessarily incomplete because of tumor extension along the bladder wall near the bladder neck and the patient’s limited operative reserve, as described in several reported bladder lymphoma cases in which TURBT serves primarily as a diagnostic rather than definitive therapeutic procedure [4,5].

Given her ECOG (3-4) status, extensive neurologic comorbidities, and poor baseline prognosis, standard anthracycline-based chemoimmunotherapy was not appropriate; instead, she received rituximab monotherapy followed by involved-site radiotherapy, achieving clear metabolic and anatomic regression of the bladder lesion on PET/CT without early evidence of progression. Ongoing surveillance and intervention must be tempered by her severe functional impairment and guarded long-term outlook, even in the absence of active lymphoma.

Compared with published bladder DLBCL series, this patient fits the usual epidemiologic pattern of an elderly female with localized Stage IE disease but differs in presentation and management. Most reported cases present with painless macroscopic hematuria and irritative LUTS [4,5,8], whereas this case was dominated by sepsis and obstructive uropathy, highlighting how bladder lymphoma can be masked by common urologic and infectious conditions. Standard management in the literature favors R-CHOP-based regimens with or without consolidative radiotherapy after diagnostic TURBT, with excellent remission rates and favorable survival in localized disease [4,6].

In contrast, the use of rituximab plus radiotherapy in this case, driven by extreme frailty and competing mortality risks, represents a de-escalated strategy that is rarely described in primary bladder DLBCL. While this approach achieved meaningful short-term disease control, this observation is based on a single case with only eight months of follow-up and should not be interpreted as evidence of a curative or generalizable treatment strategy; longer follow-up and additional cases are needed before conclusions can be drawn about durability or broader applicability in similarly frail patients [4].

Literature review

This narrative review was informed by a literature search of PubMed for case reports and small case series describing primary DLBCL of the urinary bladder, published in English through February 2026. Records were identified using the search terms “lymphoma” OR “DLBCL” combined with “bladder,” restricted to the title or abstract fields, supplemented by review of reference lists of relevant articles. Reports were included when DLBCL was the histologically confirmed, dominant site of disease within the bladder at presentation (primary bladder DLBCL); cases with clearly secondary or systemic bladder involvement at diagnosis were excluded unless the bladder was the presenting and dominant site of disease. 14 cases meeting these criteria were identified and are summarized in Table 3, alongside the present case.

As noted in the Introduction, DLBCL is subdivided into GCB and ABC/non-GCB molecular subtypes with distinct prognostic implications [2,3]. R-CHOP (rituximab, cyclophosphamide, doxorubicin, vincristine, and prednisolone) remains the standard first-line regimen, inducing remission in approximately 60-70% of patients, though outcomes are significantly worse in double-hit lymphomas (concurrent MYC/BCL2 and/or BCL6 rearrangements) or double-expressor phenotypes (MYC and BCL2 protein overexpression) [1,3,11].

The first description of bladder lymphoma was reported by Eve and Chaffey in 1885 [7,8]. Contemporary reviews incorporating multi-database searches have identified over 100 cases of primary bladder lymphoma in aggregate, though primary bladder DLBCL specifically remains particularly rare [6,7,8].

Epidemiologically, primary bladder DLBCL primarily affects older adults, with a median age of 68 years (range 35-89) and a notable female predominance [4,6]. A pooled analysis by Wang et al. indicated a mean age of 68 years, with 69% of patients being female [6,10]. Clinically, the most common presenting symptom is painless macroscopic hematuria, followed by irritative LUTS such as frequency, urgency, nocturia, and dysuria [5,6,8]. Some patients may present primarily with LUTS without evident hematuria, potentially resulting in misdiagnosis as benign prostatic hyperplasia or chronic cystitis, as highlighted in various case reports [7,9]. Table 3 provides a comprehensive summary of the clinical, pathological, and treatment outcome data from the 14 primary bladder DLBCL cases included in this review.

**Table 3:** Summary of included primary bladder DLBCL cases - clinical features and treatment outcomes (n = 14, sorted by year of publication) Stage IE denotes a single extranodal site with no nodal involvement (Ann Arbor staging). Case 3 represents a centroblastic high-grade BCL classified as DLBCL on morphology. †The patient reported by Xu et al. [20] underwent open radical cystectomy with bilateral cutaneous ureterostomy; planned postoperative R-CHOP was not administered owing to early postoperative death. CHOP: Cyclophosphamide + doxorubicin + vincristine + prednisolone; NR: Not reported; TURBT: Transurethral resection of bladder tumor; CHEP: Cyclophosphamide + epirubicin + vincristine + prednisolone; R-CHOP: Rituximab + cyclophosphamide + doxorubicin + vincristine + prednisolone; IVA: ; R^2^: Rituximab + lenalidomide; EGFR-TKI: Epidermal growth factor receptor tyrosine kinase inhibitor; R-DA-EPOCH: Rituximab + dose-adjusted etoposide + prednisolone + vincristine + cyclophosphamide + doxorubicin; DLBCL: Diffuse large B-cell lymphoma; BCL: B-cell lymphoma

Study	Age/Sex	Stage	Tumor Extent	Treatment Regimen	Radiotherapy	Surgery
Tasu et al. [12] - Case 2	68M	Stage IE	Solitary sessile mass, lateral wall; confined to mucosa and submucosa	CHOP	NR	TURBT
Tasu et al. [2] - Case 3	70M	Stage IE	Two masses on posterior bladder wall	Mitoxantrone + ifosfamide × 6 cycles	No	TURBT
Coskun et al. [13]	73F	NR	Large mass filling entire bladder; left ureteric orifice involvement	CHEP	No	TURBT
Hayashi et al. [14]	75F	Stage IVA	Diffuse bladder wall thickening; bilateral hydronephrosis	R-CHOP × 3 cycles (dose-reduced: age and renal dysfunction)	No	Not applicable
Simpson et al. [9]	48M	NR	4.9 × 6.3 cm, right anterolateral wall; muscularis propria invasion	R-CHOP × 6 cycles	No	TURBT
Deel et al. [15]	83M	NR	Muscularis propria, prostate, and urethra invasion	TURBT; no systemic chemotherapy	No	TURBT
Wang et al. [6]	62M	NR	4.7 × 3.0 cm; bilateral seminal vesicle involvement	R-CHOP × 6 cycles	No	TURBT
Ansari et al. [16]	64M	Stage IE	Diffuse bladder wall involvement	R-CHOP × 6 cycles + radiotherapy (36 Gy)	Yes	TURBT
Xu et al. [17]	77F	Systemic (lung + bladder)	8.0 × 3.4 cm, left posterior wall; left ureteral involvement	R² × 6 cycles; continued EGFR-TKI for concurrent lung primary	No	Diagnostic cystoscopy/biopsy
Sain et al. [18]	>80F	NR	Posterior wall mass; bilateral hydroureteronephrosis	TURBT + double-J stent only (patient declined active oncological treatment)	No	TURBT + double-J stent
Xin et al. [19]	83F	NR	22 × 15 mm, right trigone; lamina propria invasion	TURBT + R-CHOP × 6 cycles	No	TURBT
Li et al. [10]	50F	Stage IE	33 × 20 mm, left posterior wall	Transurethral cystectomy + R-CHOP × 4 cycles	No	Transurethral cystectomy
Salinas Mendoza et al. [7]	53M	NR	Multiple lesions; left lateral wall and bladder floor	R-DA-EPOCH (2 cycles administered)	No	TURBT (clot evacuation)
Xu et al. [20]	83M	Stage IV	6.3 × 9.5 × 6.8 cm; perivesical fat invasion	Radical cystectomy†; planned R-CHOP not administered	No	Radical cystectomy†

The pathophysiology of primary bladder lymphoma is predominantly conjectural. Since the bladder lacks intrinsic lymphoid tissue, it is posited that chronic cystitis or recurrent urinary infections may result in the development of acquired MALT, which could later undergo malignant transformation [4,8]. Although EBER ISH was negative in this case, the Epstein-Barr virus (EBV) has been associated with certain cases, particularly in the elderly, where it correlates with a more aggressive clinical course [11,18]. Bladder DLBCL characteristically exhibits robust expression of CD20, CD79a, and paired box gene (PAX5), together with frequent BCL2 positivity and elevated proliferation indices (Ki-67 >70%) [4,5]. Recent investigations have utilized genetic subclassification through GCB and non-GCB/ABC phenotypes, revealing that non-GCB variations exhibit more aggressive clinical behavior [3,4,9]. Bladder DLBCL frequently has a high Ki-67 index and overexpression of MYC and BCL2, correlating with unfavorable prognoses [4].

Beyond the classic B-cell phenotype (CD20, CD79a, PAX5), some bladder DLBCLs aberrantly express epithelial markers such as p63, a p53-homolog at 3q27-28 near BCL6. Deel et al. describe a p63-positive bladder DLBCL initially misclassified as poorly differentiated urothelial carcinoma until LCA and pan-B-cell markers confirmed lymphoma, consistent with prior series showing p63 expression in 15-70% of DLBCLs, typically with higher proliferative indices but inconsistent prognostic impact [15].

Staging and response evaluation in primary bladder DLBCL follow general lymphoma principles. Standard CT imaging of the abdomen and pelvis may not differentiate bladder DLBCL from urothelial cancer, rendering 18F-FDG PET/CT a necessary tool for precise staging and assessment of therapy efficacy [1,4]. Numerous reports on bladder DLBCL have emphasised the significance of PET/CT, especially in illustrating the metabolic confinement of the disease to the bladder and in confirming complete metabolic remission post treatment [6,9]. Dual-phase PET/CT utilizing diuretic regimens enhances imaging sensitivity by mitigating interference from urine tracer activity [10]. Bone marrow analysis, abdominal and pelvic imaging, and systemic staging can have important implications for determining whether the disease is confined to the bladder or if there is extravesical dissemination [6].

Because of the rarity of this entity, no specific treatment guidelines exist for primary bladder DLBCL. However, R-CHOP chemotherapy remains the most widely used regimen and has shown good efficacy, particularly in Stage IE disease [4,6,9]. Radiotherapy is commonly used alongside chemotherapy for individuals with significant local involvement, and several case reports have recorded sustained remissions of bladder DLBCL with R-CHOP ± RT [8,16]. Surgical procedures like TURBT or cystectomy are generally utilized for diagnostic reasons or to address problems such as blockage or hemorrhage, rather than acting as conclusive treatment [5,9]. Recently, novel treatments, such as lenalidomide-based R² regimens and chimeric antigen receptor (CAR) T-cell therapy, have emerged for relapsed or refractory DLBCL, yielding encouraging results in select patients, but clinical experience remains limited and data specific to primary bladder DLBCL are particularly sparse [17]. Table 4 summarizes treatment modality outcomes across the 14 reviewed cases, with R-CHOP-based regimens identified as the most frequently employed approach, often combined with radiotherapy. Table 5 details complete-remission and mortality counts by specific chemotherapy regimen, reported descriptively given the small size and heterogeneity of each subgroup.

**Table 4:** Treatment modality outcomes in primary DLBCL of the urinary bladder (n = 14) Given the small number of cases within each subgroup, calculated response rates are not presented; results are reported as raw case counts only, consistent with the descriptive, retrospective nature of the underlying single-case reports. "Died during follow-up" refers to all-cause mortality within the reported follow-up period. Emergency or palliative-intent procedures (nephrostomy, DJ stent insertion) were categorized under the primary treatment modality. †Xu et al. [20] underwent open radical cystectomy with bilateral cutaneous ureterostomy rather than TURBT; ‡Deel et al. [15] remained alive at 18 months on watchful waiting following diagnostic TURBT; complete remission was not documented. CR: Complete remission (no evidence of disease on clinical assessment and/or imaging at last follow-up); TURBT: Transurethral resection of bladder tumor; DLBCL: Diffuse large B-cell lymphoma

Treatment Modality	n Cases	Study	CR	Died During Follow-up
Surgery alone	3	Deel et al. [15]‡, Xu et al. [20]†, Sain [18]	0	2
Chemotherapy alone	5	Hayashi et al. [14], Wang et al. [6], Simpson et al. [9], Tasu et al. - Case 3 [12], Xu et al. [17]	5	0
Chemotherapy + Radiotherapy	1	Ansari et al. [16]	1	0
Chemotherapy + Surgery	4	Coskun et al. [13], Li et al. [10], Salinas Mendoza et al. [7], Xin et al. [19]	2	2
Chemotherapy + Radiotherapy + Surgery	1	Tasu et al. - Case 2 [12]	1	0
Total	14		9	4

**Table 5:** Chemotherapy regimen outcomes in primary DLBCL of the urinary bladder (n = 11) Given the small number of cases within each regimen group, calculated response rates are not presented; results are reported as raw case counts only. Three cases received no systemic chemotherapy (Deel et al. [15], Xu et al. [20], and Sain et al. [18]) and are therefore excluded. *Tasu et al. - Case 2 [12] received CHOP and achieved CR. The disease relapsed at five months, and a second complete remission was achieved with CHOP plus radiotherapy. CR: Complete remission; R-CHOP: Rituximab + cyclophosphamide + doxorubicin + vincristine + prednisolone; CHOP: Cyclophosphamide + doxorubicin + vincristine + prednisolone; CHEP: Cyclophosphamide + epirubicin + vincristine + prednisolone; R-DA-EPOCH: Rituximab + dose-adjusted etoposide + prednisolone + vincristine + cyclophosphamide + doxorubicin; R^2^: Rituximab + lenalidomide; DLBCL: Diffuse large B-cell lymphoma

Chemotherapy Regimen	n Cases	Study	CR	Died During Follow-Up
R-CHOP (standard dose)	5	Ansari et al. [16], Li et al. [10], Wang et al. [6], Xin et al. [19], Simpson et al. [9]	5	0
R-CHOP (half-dose)	1	Hayashi et al. [14]	1	0
CHOP (without rituximab)	1	Tasu et al. - Case 2 [12]*	1	0
CHEP (without rituximab)	1	Coskun et al. [13]	0	1
R-DA-EPOCH	1	Salinas Mendoza et al. [7]	0	1
Mitoxantrone + Ifosfamide	1	Tasu et al. - Case 3 [12]	1	0
R²	1	Xu et al. [17]	1	0
Total	11		9	2

Among the 14 cases summarized in Table 3, Stage IE disease managed with R-CHOP has been associated with notable one-year and five-year survival rates (exceeding 90% and 60-70%, respectively). Conversely, cases with advanced-stage disease, extravesical extension, or adverse biological features (non-GCB phenotype or EBV-positive status) demonstrate markedly reduced survival [4,9].

## Conclusions

Primary DLBCL of the urinary bladder is a rare and diagnostically challenging malignancy that frequently resembles more prevalent urological conditions. Timely diagnosis with cystoscopy, immunohistochemistry, and PET/CT is essential for accurate staging and treatment. R-CHOP continues to be the standard treatment; however, novel agents including R² and CAR T-cell therapy present potential alternatives for refractory cases. Tables 3, 4, and 5 collectively summarize clinical characteristics, diagnostic methods, therapeutic approaches, and treatment outcomes from a representative subset of 14 documented primary bladder DLBCL cases identified through our literature search, rather than the majority of cases reported worldwide. This case highlights the importance of tailoring surveillance and interventions according to the patient’s functional status and comorbidities for rare extranodal lymphomas like primary bladder DLBCL. The findings and treatment approach described here are descriptive and hypothesis-generating rather than definitive; they should not be generalized beyond similarly frail, carefully selected patients without further supporting evidence. It underscores the need for more comprehensive data and registry-based initiatives to better define effective, risk-adapted therapies for frail individuals with this condition.
